# Early prediction of resistance to tyrosine kinase inhibitors by plasma monitoring of *EGFR* mutations in NSCLC: a new algorithm for patient selection and personalized treatment

**DOI:** 10.18632/oncotarget.27517

**Published:** 2020-03-17

**Authors:** Fiamma Buttitta, Lara Felicioni, Alessia Di Lorito, Alessio Cortellini, Luciana Irtelli, Davide Brocco, Pietro Di Marino, Donatella Traisci, Nicola D’Ostilio, Alessandra Di Paolo, Francesco Malorgio, Pasquale Assalone, Sonia Di Felice, Francesca Fabbri, Giovanni Cianci, Michele De Tursi, Antonio Marchetti

**Affiliations:** ^1^Laboratory of Diagnostic Molecular Oncology, Center for Advanced Studies and Technology (CAST), University of Chieti, Chieti, Italy; ^2^Department of Medical and Oral Sciences and Biotechnologies, University of Chieti, Chieti, Italy; ^3^Department of Pathology, SS Annunziata Clinical Hospital, Chieti, Italy; ^4^Department of Oncology, San Salvatore Hospital, L’Aquila, Italy; ^5^Department of Oncology, SS Annunziata Clinical Hospital, Chieti, Italy; ^6^Department of Oncology, Floraspe Renzetti Hospital, Lanciano, Italy; ^7^Department of Oncology, Spirito Santo Hospital, Pescara, Italy; ^8^Department of Oncology, “S.S. Giovanni Paolo II” Veneziale Hospital, Isernia, Italy; ^9^Department of Oncology, Giuseppe Mazzini Hospital, Teramo, Italy

**Keywords:** epidermal growth factor receptor (*EGFR*), tyrosine-kinase Inhibitors, circulating tumor-DNA (ct-DNA), massive parallel sequencing (MPS), resistance-inducing mutation

## Abstract

In Non-Small-Cell Lung Cancer (NSCLC) patients treated with Tyrosine Kinase-Inhibitors (TKIs) therapy, the emergence of acquired resistance can be investigated by plasma monitoring of circulating tumor DNA (ctDNA). A series of 116 patients with *EGFR*-positive lung adenocarcinomas were treated with first/second generation *EGFR* TKIs. At clinical progression, 64 (55%) *EGFR* T790M plasma positive patients were subjected to second line-treatment with osimertinib and strictly monitored during the first month of therapy. Plasma analysis by the *EGFR* Cobas test showed in 57 (89%) cases a substantial decrease in the levels of the sensitizing *EGFR* mutant allele (s*EGFR*ma), down to a not detectable value. These patients were defined as plasmatic good responders (PGR). In 7 (11%) patients, the s*EGFR*ma did not drop to zero (plasmatic poor responders, PPR). In these latter cases, Massive Parallel Sequencing (MPS) analysis at the end of the first month and at clinical progression showed the presence of resistant-inducing mutations, including MET and HER2 gene amplification, KRAS and PIK3CA gene mutations. PPR showed disease progression in 5 (71%) cases, stable disease in 2 (29%) cases, and a shorter median Progression-free survival (PFS) (4.3 ± 1.1 months) than that observed in PGR (13.3 ± 1.2 months) (*P* < 0.0001). Our data indicate that plasma monitoring by a simple RT-PCR-based *EGFR* mutation test in the first month of treatment may be useful for a rapid identification of patients to be subjected to further characterization by MPS. A diagnostic algorithm for an early detection of resistance-inducing mutations and patient management is reported.

## INTRODUCTION

*EGFR* mutations that confer sensitivity to tyrosine kinase inhibitors (TKI) in non-small cell lung carcinomas paved the way for a precision medicine approach in this neoplastic form. At the time of clinical diagnosis, the detection of *EGFR* mutations in tumor tissue or ctDNA, when tissue is unavailable, is mandatory for the selection of patients [1, 2]. Several studies have shown a high concordance between the presence of *EGFR* mutations in tissue and plasma, especially in patients with diffuse metastatic disease [3, 4]. A limited number of studies conducted with quantitative of semi-quantitative mutation detection methods have suggested a correlation between the amount of the sensitizing *EGFR* mutant allele (s*EGFR*ma) in plasma and tumor burden [5–7].

In a previous study, we have shown for the first time that NSCLC patients carrying *EGFR* mutations in tumor tissue and subjected to first-line treatment with *EGFR* TKIs, can be strictly monitored in the first days of treatment by repeated blood draws to quantify *EGFR* mutant alleles in plasma [5]. This accurate quantification of *EGFR* mutations has proven to be useful for early prediction of clinical response. A rapid decrease of the s*EGFR*ma in plasma, identified a category of patients defined “rapid plasma responders” and was associated with higher levels of tumor shrinkage at 2 months, evaluated by RECIST criteria. On the other hand, a slow decrease of *EGFR* mutant alleles in plasma was typical of “slow plasma responders” a patient category who showed lower overall response rates (ORRs). These results strongly suggest that the amount of *EGFR* sensitizing mutations in plasma reflects the tumor burden and that the fluctuations in the levels of *EGFR* sensitizing mutations in plasma are closely related to tumor load variations [4–8].

Most patients treated with first/second generation TKI inhibitors do eventually progress after a median of 9–14 months as a consequence of mutational changes responsible for acquired resistance [9–13]. Among these changes, the *EGFR*-T790M mutation represents one of the most frequent resistance mechanisms at progression (about 50–60% of cases) [14–18] and is the target of a pharmacological approach with osimertinib, a third generation *EGFR* TKI [19–21].

However, after a medium period of about 12 months, even patients subjected to second line treatment with osimertinib, develop resistance with various mechanisms, including *EGFR* SNV, MET and HER2 amplification, genetic fusions etc. Recently, a series of clinical trials led to the approval of first line treatment of *EGFR*-positive NSCLC patients with osimertinib [22, 23]. In these studies a mean PFS of 19 months and similar tumor resistance mechanisms have been reported.

The emergence of acquired resistance in patients receiving TKI therapy can be investigated by plasma monitoring of circulating tumor DNA (ctDNA), as shown in a series of previous studies by our group and others [4–8]. The assessment of the molecular mechanisms involved in the acquisition of resistance requires the use of complex and expensive multi-gene tests, mainly based on massive parallel sequencing (MPS), now also available for analysis of plasma samples. An early detection of the mutation/s involved in the resistance mechanism may be important to further select patients for specific treatments. In the present study, we report a diagnostic algorithm for an early detection of resistance-inducing mutations in *EGFR*-TKI treated patients.

## RESULTS

**Figure 1:**
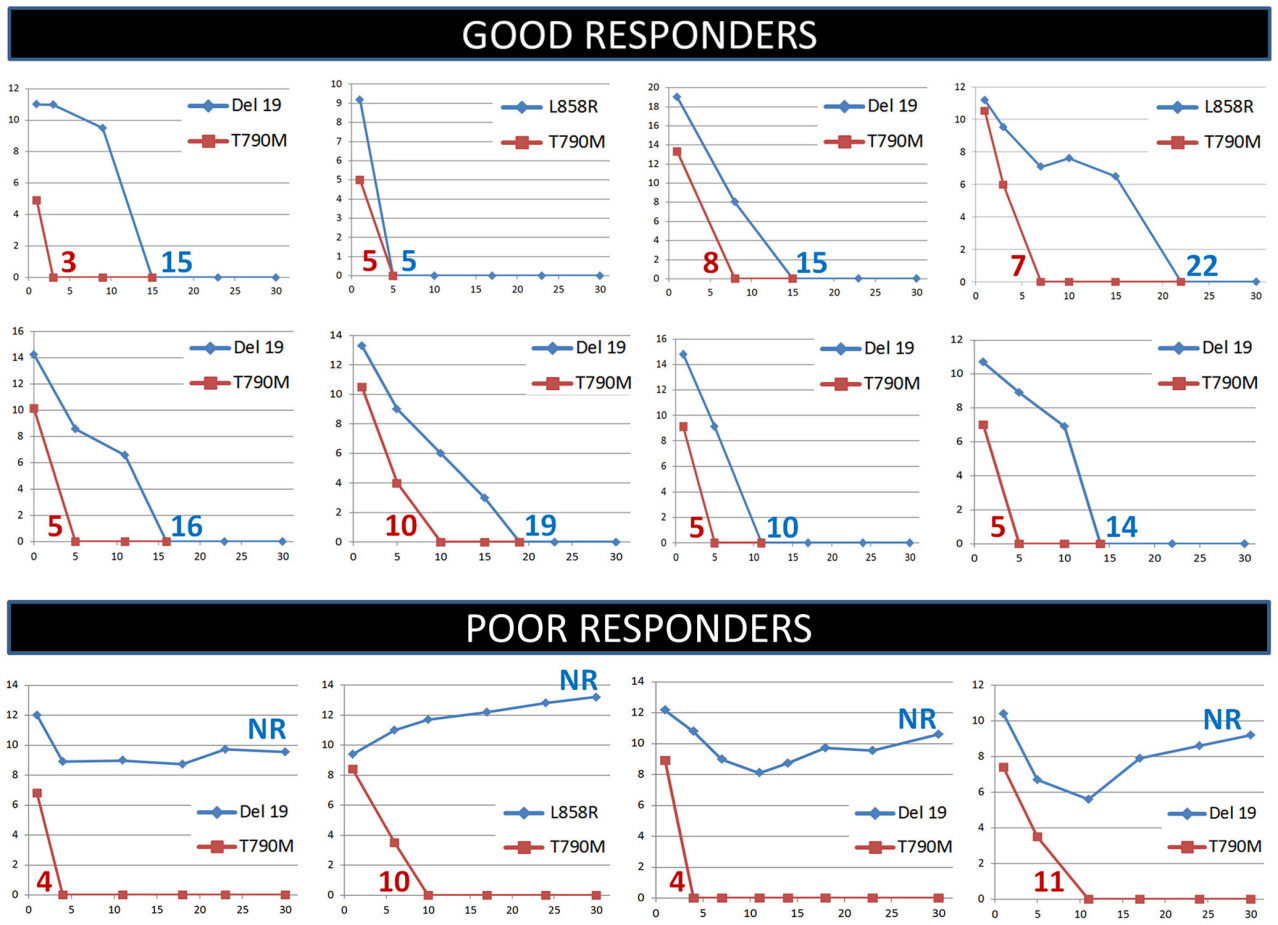
Monitoring of the EGFR sensitizing (blue curves) and T790M (red curves) mutations in plasma during the first month of treatment with osimertinib in non-small cell lung cancer patients. Representative cases of good and poor responders are shown. Numbers indicate clearing times for the EGFR sensitizing mutation (blue) or the T790M mutation (red). NR, clearing not reached.

A series of 116 patients with *EGFR*-positive lung adenocarcinomas were treated with first/second generation *EGFR* TKIs. At clinical progression, 64 (55%) patients were found to be positive for the T790M mutation, detected in liquid biopsies or tissue rebiopsies, and hence subjected to second line treatment with osimertinib. Clinicopathological data, type of sensitizing *EGFR* mutations detected in tissue and plasma, and type of first line *EGFR* TKI used in this series are reported in Table 1. The 64 patients treated with osimertinib were strictly monitored during the first month of therapy by plasma analysis of the *EGFR* gene status at short intervals (3–8 days, mean 5.7 days) (Figure 1), for a total of 322 plasma tests.

**Table 1:** Clinicopatological and genetic features of a series of NSCLC patients treated with first/second generation TKIs before selection for osimertinib tretment

No. of patients (%) total 64	
**Gender**	
**Female**	**48 (75)**
**Male**	**16 (25)**
**Age (years)**	
**Median**	**63.5 ± 7.2**
**Range**	**(52–78)**
**Baseline SNC metastases**	
**No**	**44 (68.7)**
**Yes**	**20 (31.3)**
**Primary EGFR mutation (plasma)**	
**Exon 19 - Deletion**	**47 (73.4)**
**L858R**	**17 (26.6)**
**First line EGFR TKi**	
**Gefitinib**	**47 (73.4)**
**Erlotinib**	**5 (7.8)**
**Afatinib**	**8 (12.5)**
**Dacomitinib**	**4 (6.3)**

Plasma analysis, conducted by the *EGFR* Cobas test, showed in 57 (89%) of the 64 cases a rapid decrease in the levels of the s*EGFR*ma, down to a value not detectable by the test, within the first month of treatment. The time at which the s*EGFR*ma reached an undetectable level in plasma (s*EGFR*ma clearing time) was recorded in each case. The mean clearing time for the s*EGFR*ma was 17.18 days. A similar analysis, conducted on the T790M *EGFR* mutant allele, revealed a mean clearing time of 7.62 days. The difference between the two values was statistically significant (*P* < 0.0001). The 57 patients in which plasma was cleared of the s*EGFR*ma within the first month of therapy were defined as plasmatic good responders. In 7 (11%) of the 64 patients, a rapid clearing of the T790M *EGFR* mutant allele was observed, while the s*EGFR*ma, after a slight and brief decrease (6 cases) or in the absence of an initial decrement (a single case), remained high during the first month of treatment. These 7 patients were designed as plasmatic poor responders, since the s*EGFR*ma in plasma was not cleared within the first month (Figure 1).

**Figure 2:**
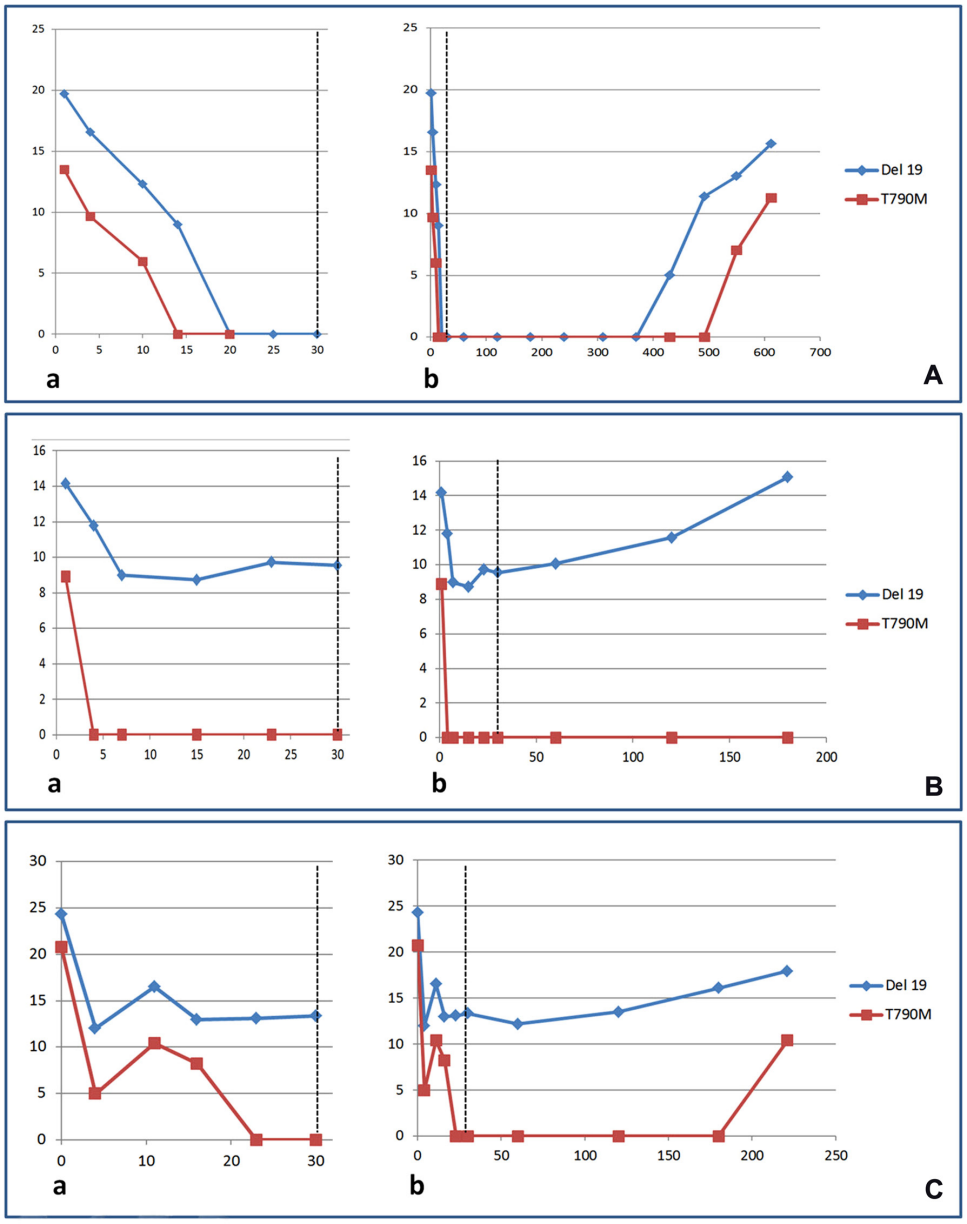
Monitoring of the EGFR sensitizing (blue curves) and T790M (red curves) mutations in plasma during the first month of treatment (**a**) or during the total period of treatment (**b**) with osimertinib in non-small cell lung cancer patients. Representative cases of good (**A**) and poor responders (**B**, **C**) are shown.

After the first month of treatment with osimertinib, all patients underwent *EGFR* plasma monitoring at the (Figure 2) end of the second month and then every two months. A reemergence of the s*EGFR*ma after clearing (in good responders) (Figure 2Ab) or a progressively increasing level of the s*EGFR*ma (in poor responders) (Figure 2Bb–2Cb), indicating a plasmatic progression, was observed in all but 15 cases. Nine of these 15 cases had a short (less than 6 months) follow-up. Plasma progression occurred on average at 276 (± 55) days from treatment initiation with osimertinib. The T790M allele reemerged in plasma, constantly after the s*EGFR*ma, in 33 (52%) of cases.

At the time of clinical progression all the poor responders were subjected to further plasma withdrawal for MPS analysis that showed the presence of a series of genomic alterations which have been reported as molecular events inducing resistance to osimertinib treatment, including *MET* gene amplification in 3 (43%) cases, *HER2* gene amplification, *EGFR*, *KRAS*, *BRAF*, and *PIK3CA* mutations. MPS analysis was repeated in these cases on plasma samples collected at the end of the first month of treatment with osimertinib with similar results in terms of resistant inducing mutations, as reported in Table 2. In addition, we tested by mps a group of six good responders during the first month of treatment with osimertinib. In these cases, no pathogenetic or known resistant-inducing mutations were detected.

**Table 2:** Genomic alterations detected in plasma samples of non-small cell lung cancer patients in treatment with osimertinib

Case	Gene alterations detected at the end of the first month of treatment	Gene alterations detected at clinical progression
**1**	***EGFR*, L858R **; *Met* amplification	***EGFR*, L858R **, *Met* amplifiction
**2**	***EGFR*, Ex19 Deletion **; *EGFR* T790M; *HER2* amplification	***EGFR*, Ex19 Deletion **; *EGFR* T790M; *HER2* amplification
**3**	**E*GFR* Ex19 Deletion**; *Met* amplification; *TP53*, p. G245D	***EGFR* Ex19 Deletion **; *Met* amplification; *TP53*, p. G245D; *TP53*, p. C141W
**4**	**EGFR EX 19 Deletion**; *EGFR*, T790M; *Met* amplification	**EGFR EX 19 Deletion**; *EGFR*, T790M; *Met* amplification
**5**	**EGFR EX 19 Deletion**; *EGFR* T790M; *EGFR*, C797S	**EGFR EX 19 Deletion**; *EGFR* T790M; *EGFR*, C797S; *BRAF* V600E
**6**	***EGFR*, L858R; ** *EGFR* T790M; *Kras*, G12D	***EGFR*, L858R; ** *EGFR* T790M; *Kras*, G12D
**7**	***EGFR* EX 19 Deletion **; *PIK3CA, E545K*	***EGFR* EX 19 Deletion **; *PIK3CA, E545K*

The primary EGFR sensitizing mutation is reported in bold.

In terms of objective responses, according to RECIST criteria, the plasmatic poor responders showed disease progression in 5 (71%) cases and stable disease in 2 (29%) cases, no partial responses were seen in this patient category. The percentage of patients with disease progression, stable disease, and partial response in poor responders was significantly different (*P* < 0.0001) from that observed in plasmatic good responders, see Table 3.

**Table 3:** Correlation between mutated plasma *EGFR* response and clinical response evaluated by RECIST criteria

Plasma EGFR Response	Clinical Response			
	**Progressive disease**	**Stable disease**	**Partial response**	**TOTAL**
Poor responder	5 (71.4%)	2 (28.6%)	0 (0%)	7 (100%)
Good responder	3 (5.3%)	15 (26.3%)	39 (68.4%)	57 (100%)
TOTAL	8 (12.5%)	17 (26.6%)	39 (60.9%)	64 (100%)

**Figure 3:**
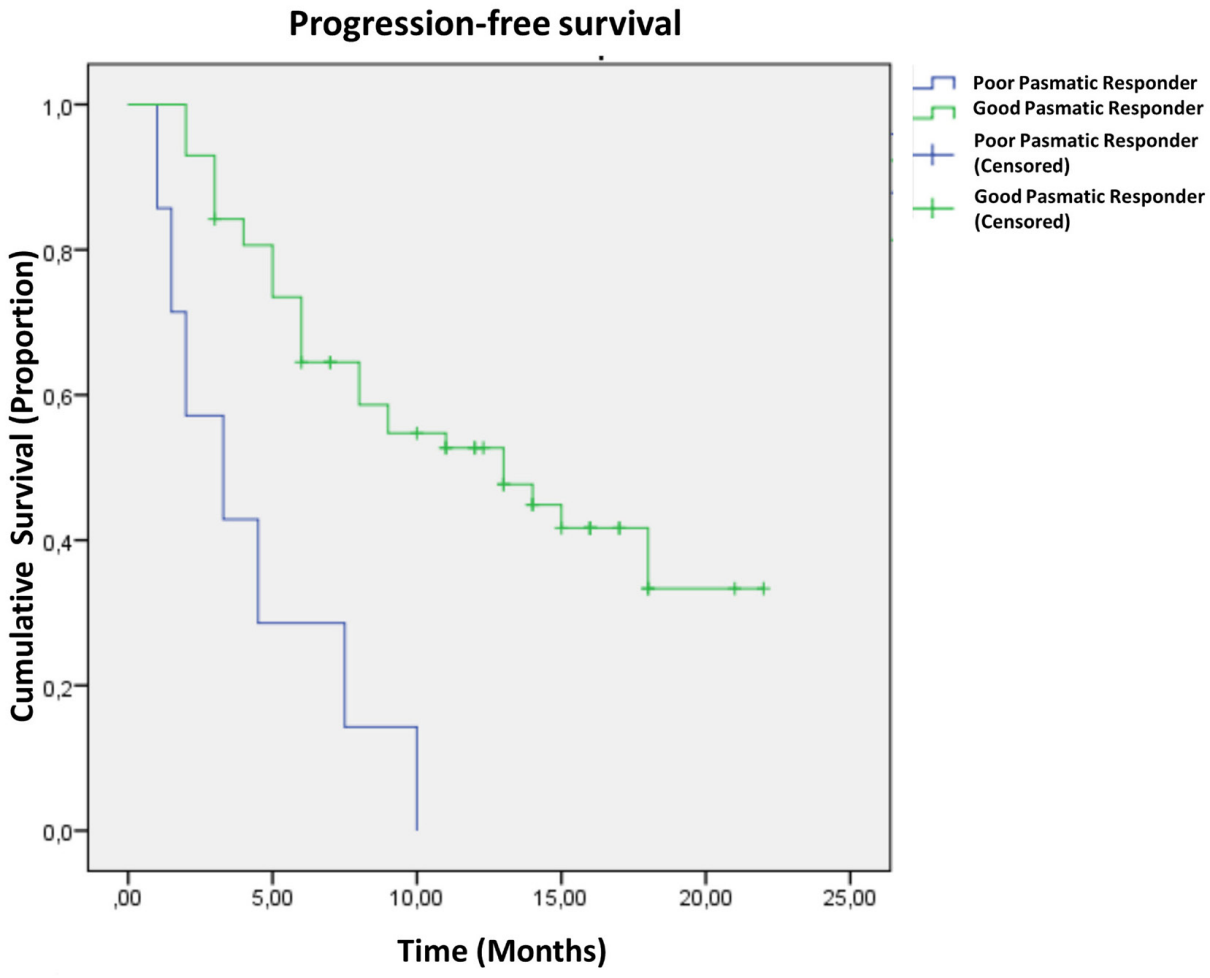
Progression-free survival curves in 64 patients with non-small cell lung carcinoma based on the plasmatic response to osimertinib treatment. Curve differences are statistically significant (see text for details).

Median clinical follow-up for this population of patients was 13 months after the beginning of treatment with osimertinib. Median PFS was 12.3 months (95% CI, 10.8 to 15.2). Survival curves (Figure 3), estimated using the method of Kaplan and Meier, revealed that patients with plasmatic poor response had shorter median PFS (4.3 ± 1.1 months) than that observed in plasmatic good responders (13.3 ± 1.2 months) (*P* < 0.0001). In these latter patients plasma progression, defined as the time of reappearance of the s*EGFR*ma in plasma, preceded clinical progression, on average, of 93 ± 21 days.

## DISCUSSION

In this study on NSCLC patients who received second line osimertinib after a first-line TKIs we have shown that a strict monitoring of the s*EGFR*ma in plasma during the first month of treatment may identify a subset of early-resistant patients carrying molecular alterations potentially treatable with specific drugs.

The results of one of our previous studies, in keeping with a series of data emerged in the literature, indicate that an accurate quantification of the s*EGFR*ma in plasma reflects tumor burden [5, 6] and that variations of this parameter can be used to monitor the effectiveness of treatment with TKIs. This is due to the fact that sensitizing mutations are driver events diffusely present in neoplastic cells as a result of clonal expansion. This makes tumors dependent on these mutations and is the basis of molecular target therapies. The sensitizing mutation is therefore to be considered as the main genetic hallmark of the tumor that can be rapidly and easily detected and quantified in plasma, providing useful indications for treatment.

It is widely accepted that an increase (after clearing) in the plasma level the of s*EGFR*ma during treatment with TKIs is an early sign of tumor resistance [5, 6]. Our data indicate that the non-clearing of the s*EGFR*ma in plasma during the first month of treatment can also be useful to immediately recognize an intrinsically resistant tumor or a tumor potentially bearing an early acquired resistance. This initial characterization, requiring only a few plasma assessments by a simple and inexpensive mutation test, may be easily introduced in the clinical practice to rapidly select patients with resistant tumors (poor responders). In these patients, an immediate evaluation of resistance-inducing mutation/s by massive parallele sequencing is then important to establish the mutational cause of the resistance and how to adapt the treatment accordingly.

A number of additional observations in this study need to be further discussed for their potential clinical implications. The strict monitoring of patients during the first month of treatment with osimertinib has allowed to accurately define the effect of this therapy on the plasmatic levels of both the sensitizing and resistance-inducing, T790M mutation. The clearing time for the T790M *EGFR* mutant allele was significantly shorter than that observed for the *EGFR* s*EGFR*ma. This could be in part ascribed to the lower clonal expansion of the T790M mutation in tumors at the time of progression, in part to the higher efficacy of osimertinib on cells carring the T790M mutant allele. Paradoxically, in diagnostic terms, at the beginning of treatment with osimertinib, the rapid decrement in the levels of the T790M mutant allele observed in all cases can be considered as an early predictor of sensitivity of the T790M positive tumor clones. On the other hand, the increasing levels of the s*EGFR*ma are an early sign of tumor resistance. Another interesting point is that the slopes of the curves and clearing times observed for the s*EGFR*ma in good responders are reminiscent of those we previously reported in good responders to first line therapy with first generation TKIs [5], in line with the high clinical efficacy of osimertinib even in second line treatments.

In good responders, the s*EGFR*ma in plasma was cleared within the first month and remained undetectable until its reemergence at variable times before clinical progression (about 3 months on the average). This phenomenon has been observed in all treated patients and may be useful to activate a closer follow-up after the reemergence of the s*EGFR*ma in plasma.

Regarding the poor responders, in all but one cases s*EGFR*ma plasmatic values during the first month of treatment showed an initial slight and brief decrease, almost correspondent to the reduction of the T790 mutant allele. Immediately after, the levels of the s*EGFR*ma progressively increased in all cases. In both good and poor responders, the levels of the T790M could, in parallel, follow the progressive increment of the s*EGFR*ma or not, supporting the hypothesis that the mechanism of secondary resistance occurs in the same clone carrying the T790M mutation or not, respectively.

In addition, we have shown that the overall response rate and progression–free survival was significantly worse in poor responders. Our data indicate that a simple plasma monitoring during the first month of treatment can identify subsets of patients with different clinical outcomes.

At clinical progression, all the poor responders were subjected to plasma analysis by MPS that revealed the presence of a number of mutations including *MET* and *HER2* gene amplification and *KRAS*, *BRAF*, *PIK3CA* mutations which are known to be involved in the acquisition of resistance to osimertinib. In these cases, MPS analysis was repeated on plasma samples collected at the end of the first month of treatment, showing that these resistant-inducing mutations were already present in tumor cells.

These results indicate that a rapid and easy Real-time PCR-based assay could be used during the first month of treatment with osimertinib in order to accurately monitor the patients and select those which are resistant to the drug. These latter cases, at the end of the first month of treatment with osimertinib, should be tested by plasma analysis with MPS to evaluate the resistance mechanisms activated in tumor cells and treat the patients with appropriate drugs. A number of trials have been activated to treat patients with double or multiple mutations with combined treatments. For instance, tumors carrying *EGFR* mutations and met amplification can be efficiently treated with osimertinib plus savolitinib [24–26].

**Figure 4:**
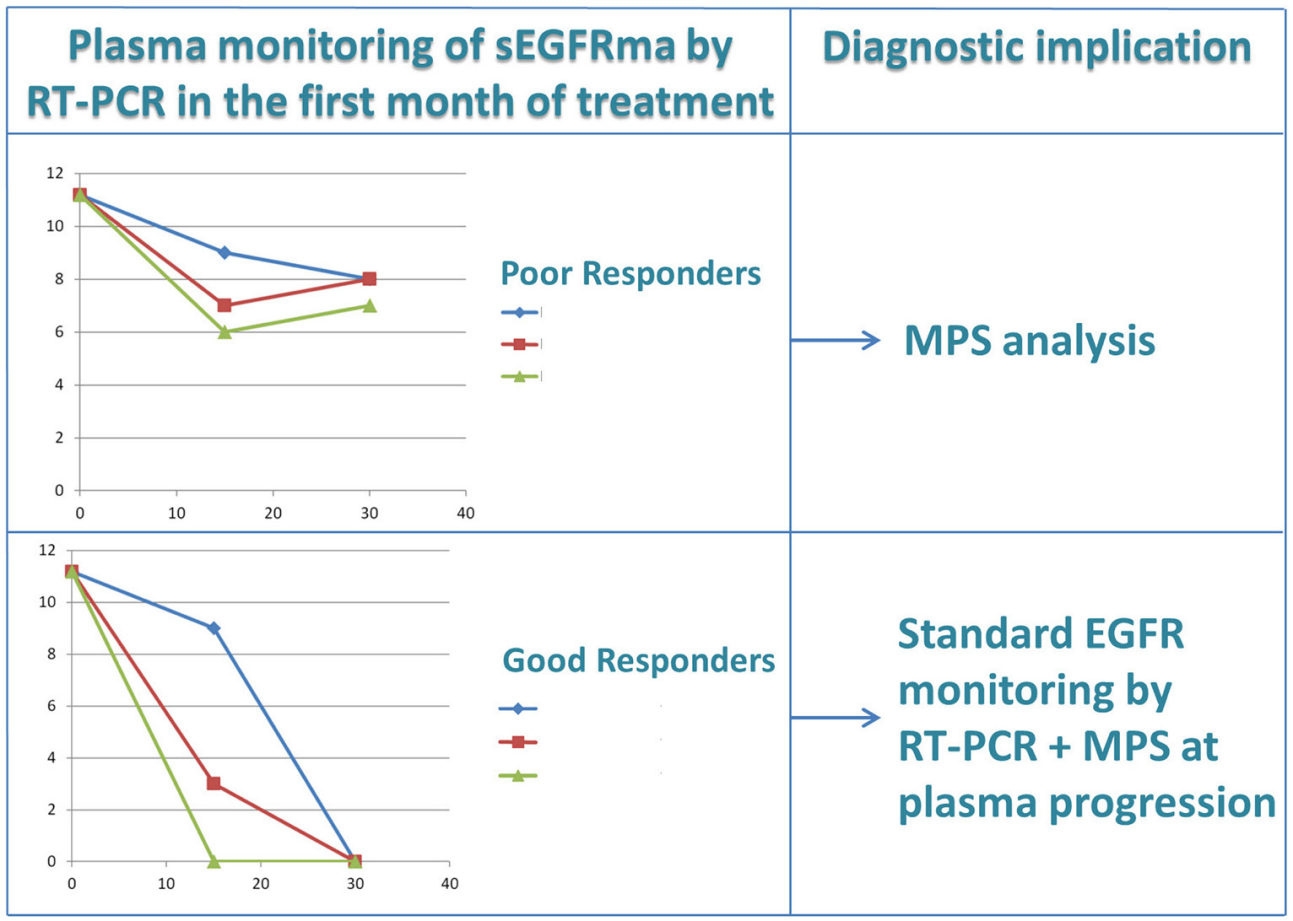
Diagnostic algorithm for early prediction of resistance to tyrosine kinase inhibitors by RT-PCR plasma monitoring of EGFR mutations in NSCLC. Simulated examples of sEGFRma plasma curves in good and poor responders are reported (see text for details). sEGFRma, sensitizing EGFR mutant allele; RT-PCR, Real-Time-Polymerase Chain Reaction.

Based on these considerations, we propose a diagnostic algorithm (see Figure 4) for patients treated with *EGFR*-TKIs, that could be applied both in first and second line of treatment, which provides two plasma assessments of the *EGFR* gene at 15 and 30 days after the baseline test by means of a quantitative or semi-quantitative Real-time PCR assay. In the case where the sensitizing mutation is not completely cleared (using the semi-quantititative cobas test) or not significantly reduced (using a quantitative method), an immediate plasma assessment by MPS is suggested in order to detect the mutation mechanism at the basis of resistance and then proceed to modify, if necessary, the treatment option. This diagnostic algorithm could also be useful for NSCLC patients treated with Osimertinib in first line. Indeed, the results of the Flaura trial indicate an overall response rate of 80% after first line treatment with osimertinib. Hince, about 20% of patients are expected to be unresponsive to treatment [23].

In conclusion, our results indicate that a subset of NSCLC patients subjected to second line treatment with osimertinib are resistant to treatment due to the presence of different types of mutations. Plasma monitoring by a simple RT-PCR-based *EGFR* mutation test in the first month of treatment may be useful to rapidly identify these cases and subject them to MPS analysis for further characterization and treatment.

## MATERIALS AND METHODS

### Patients and blood sample collection

One hundred and sixteen stage IIIB/IV NSCLC patients, carrying *EGFR* mutations in their tumors and treated with first/second generation anti-*EGFR* TKIs, were tested for T790M mutation at clinical progression by liquid biopsy or tumor rebiopsies. Sixty-four (55%) patients were proved to be T790M positive and subsequently treated with third generation *EGFR* tyrosine kinase inhibitor, osimertinib. During the first month of treatment with osimertinib, serial plasma samples were obtained at short time intervals (3–8 days), starting from the beginning of treatment (baseline). After the first month of therapy, plasma collection was repeated at the end of the second month and then every two months until clinical progression. Informed consent was obtained from all patients and the study was conducted in accordance with the precepts of the Helsinki Declaration.

### DNA extraction and assessment of EGFR mutations by RT-PCR

At each collection, two blood samples containing 5 ml were placed in BD VACUTAINER PPT K2EDTA tubes (BD Preanalytical Systems, Franklin Lakes, NJ) and subjected to plasma separation, within 30 minutes. After centrifugation at 1100 rpm for 15 minutes, plasma samples were aliquoted and stored at −80°C until DNA Extraction. DNA was extracted from 4 ml of plasma using the cobas^®^ cfDNA Sample Preparation kit vers.2 (Roche Molecular Systems, Pleasanton, CA), according to the manufacturer’s instructions and recovered in 200 μl of elution buffer. The DNA amount was not measured to avoid loss of material. Half of the volume was immediately used for *EGFR* mutation assessment by the cobas^®^ EGFR Mutation Test v2 assay (Roche Molecular Systems, Pleasanton, CA), the other half was stored for further analysis. PCR reactions were run on the cobas^®^ z 480 analyzer with *EGFR* Blood Analysis Package Software.

### Massive parallel sequencing (MPS)

An innovative MPS Approach (SureSelect Cancer All-In-One; Agilent, Santa Clara, California, USA) was used to optimize the management of the biological material. This new hybrid capture-based technology, is able to ensure, starting from DNA, high-sensitivity assessment of the mutational status of genomic regions of interest for a variety of features including not only SNVs (single nucleotide variations) and Indels (short insertions and deletions), but also CNVs (copy number variations) and fusions, by targeting translocation driver genes regardless of partner genes. NGS libraries were prepared using the SureSelectXT HS or SureSelectXT Low Input Target Enrichment system, according to the manufacturer’s instructions, and were sequenced on the Illumina Miseq/HighSeq platforms. Data were analyzed using Agilent’s SureCall software.

### Statistical analysis

The variables measured in the study were investigated for association by using the *T* test, Fisher’s exact test or *χ*2 test as appropriate. Progression-free survival (PFS) and overall survival (OS) were measured for each patient from the beginning of treatment with osimertinib. Survival curves were estimated using the Kaplan-Meier method, and differences among them evaluated by the log-rank test. A *P* value < 0.05 was considered as significant. All statistical analyses were performed using SPSS version 24.
